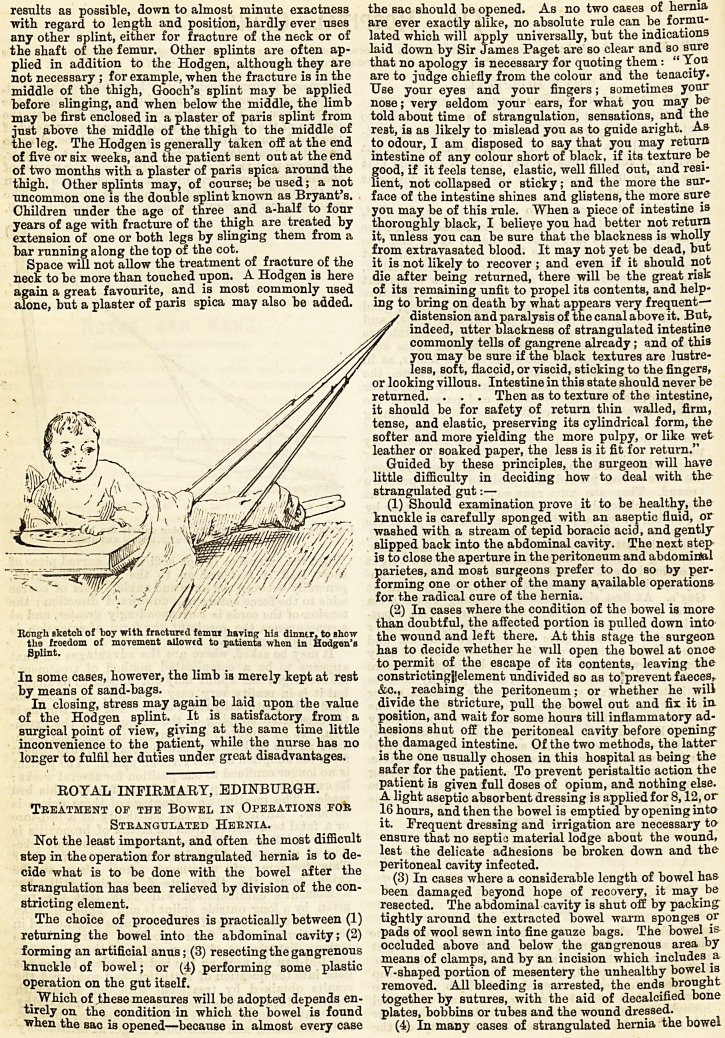# The Treatment of Fractures of the Femur

**Published:** 1893-03-11

**Authors:** 


### THE HOSPITAL CLINIC.

*[The Editor will be glad to receive offers of co-operation and contributions from members of the profession All letters should be addressed to* THE EDITOR, THE LODGE, PORCHESTER SQUARE, LONDON, W.]

### GUY'S HOSPITAL.

### THE TREATMENT OF FRACTURES OF THE FEMUR.

In the following few remarks it is not intended to enter elaborately into the numerous methods of treatment of all the varieties of fracture of the femur, but merely to refer to one or two of those methods that are in use in the treatment of the simpler forms at Guy's Hospital.

It is hardly necessary to remind the reader that, classified according to their situation, fractures of the femur are divided into those of the (1) upper end (the neck), (2) the shaft, (3) the lower end. We will first consider the treatment of the second of these varieties, namely, fracture of the shaft. The two important points to be aimed at in the treatment of a fracture are, as one's common sense suggests, firstly, good apposition of the ends of the broken bone, and secondly, continued rest in that position. Splints are designed with the object of maintaining these conditions. In fracture of the shaft of the femur, as in fracture of the shaft of any other long bone, the muscles released from tension draw up the lower fragment, so that one fractured end passes over the other. Treatment has for its object the overcoming of the action of the muscles and the readjustment of the two portions of bone to the former position. By putting tension on the muscles not only is the lower fragment drawn down, but the two ends of the bone are more or less pushed into position by the pressure around. There are numerous ways of doing this, but they all seek to obtain extension of the limb either by a pushing or by a pulling force. An example of the former is the long outside splint with a perineal band, and of the latter the simple use of a weight and pulley.

A long outside splint sometimes used with a weight extension instead of with a perineal band is not unpopular in many places, but is very rarely used at Guy's. At first sight, perhaps, nothing could seem better suited to obtain the end in view. Were it possible for the patient to become a dummy for five or six weeks, with all desire for comfort removed, and some of the ordinary physiological functions of daily life in abeyance, no method could be simpler or more satisfactory. To many, however, and especially to those patients over sixty years of age, many of whom suffer from chronic bronchitis, the forcible retention in one position is exceedingly trying, and may terminate fatally

The Hodgen splint is in very common use at Guy's, and since it is possible that some readers may not be familiar with it, we will describe it as briefly as possible. In reality it is more of an apparatus for applying extension by the aid of suspension than a splint. The splint portion of this apparatus is a strong wire frame

Hogden's Splint.

of the shape shown in the diagram. Sewn on to one side is a piece of flannel, which is afterwards brought round beneath the limb, and stitched to the other side. Before being applied, however, the ordinary stirrup used for extension is strapped to the limb. The wire frame is then placed over leg, the band of flannel brought round underneath and stitched to the other side; while the cord attached to the stirrup of the extension is firmly tied to the lower end of the splint. Extension of the limb is now obtained by slinging the splint from a T-shaped upright firmly fixed at the bottom of the bed. It is as well to raise the lower end of the bed by means of blocks. It may not be clear to some how the extension acts; a little thought, however, will show that two forces are acting; one the weight of the limb in a downward direction, the other the tension of the cords in a direction obliquely upwards. The pulling force, the resultant, acts in the line of the limb.

Leg slung in a Hodgen's Splint. Being outside the bed-clothes, it is afterwards covered with cotton wool and flannel.

When the extension is not sufficient, a curved piece of sheet lead is fastened on to the top of the splint, generally between the knee and ankle. This of course adds to the force acting in a downward direction; the tension of the cords is correspondingly greater, and as a consequence, the resultant, the force of extension, is materially increased.

It may be asked, where are the advantages of what appears to be a complicated method of treatment? At first sight the splint may not appear to be simple, but it is in reality very easy to apply, and does not need to be continually readjusted. The only trouble is the occasional slipping of the strapping of the extension. It is, however, not so much the medical attendant as the patient and the nurses who reap benefit from the adoption of this form of splint. The patient is no longer confined to one position for several weeks; he can sit up, and help to raise himself to have his bed made. The inestimable advantage of these points is obvious. Anxiety as to the possibility of a bed-sore or a fatal termination from an attack of bronchitis is removed. It may be asked, however, is not this freedom of movement deleterious to prospects of union? This freedom of movement takes place at the thigh joint, the thigh itself remains still, but a moment's consideration will show that the rest given by a long outside splint is more apparent than real. To say nothing of the fidgetiness engendered in the patient by the forced position, there must be serious disturbance of the position of the limb when the bed-pan is used or the bed is made. I have not gone into the question of statistics, and compared the results obtained by treatment with this splint with those obtained by other methods, but it is a noteworthy fact that the surgeon at Guy's who takes most interest in his fractures, and endeavours to get as satisfactory results as possible, down to almost minute exactness with regard to length and position, hardly ever uses any other splint, either for fracture of the neck or of the shaft of the femur. Other splints are often applied in addition to the Hodgen, although they are not necessary; for example, when the fracture is in the middle of the thigh, Gooch's splint may be applied before slinging, and when below the middle, the limb may be first enclosed in a plaster of paris splint from just above the middle of the thigh to the middle of the leg. The Hodgen is generally taken off at the end of five or six weeks, and the patient sent out at the end of two months with a plaster of paris spica around the thigh. Other splints may, of course, be used; a not uncommon one is the double splint known as Bryant's. Children under the age of three and a-half to four years of age with fracture of the thigh are treated by extension of one or both legs by slinging them from a bar running along the top of the cot.

Space will not allow the treatment of fracture of the neck to be more than touched upon. A Hodgen is here again a great favourite, and is most commonly used alone, but a plaster of paris spica may also be added.

Rough sketch of boy with fractured femur having his dinner, to show the freedom of movement allowed to patients when in Hodgen's Splint.

In some cases, however, the limb is merely kept at rest by means of sand-bags.

In closing, stress may again be laid upon the value of the Hodgen splint. It is satisfactory from a surgical point of view, giving at the same time little inconvenience to the patient, while the nurse has no longer to fulfil her duties under great disadvantages.

### ROYAL INFIRMARY, EDINBURGH.

### Treatment of the Bowel in Operations for Strangulated Hernia.

Not the least important, and often the most difficult step in the operation for strangulated hernia is to decide what is to be done with the bowel after the strangulation has been relieved by division of the constricting element.

The choice of procedures is practically between (1) returning the bowel into the abdominal cavity; (2) forming an artificial anus; (3) resecting the gangrenous knuckle of bowel; or (4) performing some plastic operation on the gut itself.

Which of these measures will be adopted depends entirely on the condition in which the bowel is found when the sac is opened—because in almost every case the sac should be opened. As no two cases of hernia are ever exactly alike, no absolute rule can be formulated which will apply universally, but the indications laid down by Sir James Paget are so clear and so sure that no apology is necessary for quoting them: "You are to judge chiefly from the colour and the tenacity. Use your eyes and your fingers; sometimes your nose; very seldom your ears, for what you may be told about time of strangulation, sensations, and the rest, is as likely to mislead you as to guide aright. As to odour, I am disposed to say that you may return intestine of any colour short of black, if its texture be good, if it feels tense, elastic, well filled out, and resilient, not collapsed or sticky; and the more the surface of the intestine shines and glistens, the more sure you may be of this rule. When a piece of intestine is thoroughly black, I believe you had better not return it, unless you can be sure that the blackness is wholly from extravasated blood. It may not yet be dead, but it is not likely to recover; and even if it should not die after being returned, there will be the great risk of its remaining unfit to propel its contents, and helping to bring on death by what appears very frequent—distension and paralysis of the canal above it. But, indeed, utter blackness of strangulated intestine commonly tells of gangrene already; and of this you may be sure if the black textures are lustreless, soft, flaccid, or viscid, sticking to the fingers, or looking villous. Intestine in this state should never be returned. . . . Then as to texture of the intestine, it should be for safety of return thin walled, firm, tense, and elastic, preserving its cylindrical form, the softer and more yielding the more pulpy, or like wet leather or soaked paper, the less is it fit for return."

Guided by these principles, the surgeon will have little difficulty in deciding how to deal with the strangulated gut:—

(1) Should examination prove it to be healthy, the knuckle is carefully sponged with an aseptic fluid, or washed with a stream of tepid boracic acid, and gently slipped back into the abdominal cavity. The next step is to close the aperture in the peritoneum and abdominal parietes, and most surgeons prefer to do so by performing one or other of the many available operations for the radical cure of the hernia.

(2) In cases where the condition of the bowel is more than doubtful, the affected portion is pulled down into the wound and left there. At this stage the surgeon has to decide whether he will open the bowel at once to permit of the escape of its contents, leaving the constricting element undivided so as to prevent faeces, &c., reaching the peritoneum; or whether he will divide the stricture, pull the bowel out and fix it in position, and wait for some hours till inflammatory adhesions shut off the peritoneal cavity before opening the damaged intestine. Of the two methods, the latter is the one usually chosen in this hospital as being the safer for the patient. To prevent peristaltic action the patient is given full doses of opium, and nothing else. A light aseptic absorbent dressing is applied for 8, 12, or 16 hours, and then the bowel is emptied by opening into it. Frequent dressing and irrigation are necessary to ensure that no septic material lodge about the wound, lest the delicate adhesions be broken down and the peritoneal cavity infected.

(3) In cases where a considerable length of bowel has been damaged beyond hope of recovery, it may be resected. The abdominal cavity is shut off by packing tightly around the extracted bowel warm sponges or pads of wool sewn into fine gauze bags. The bowel is occluded above and below the gangrenous area by means of clamps, and by an incision which includes a V-shaped portion of mesentery the unhealthy bowel is removed. All bleeding is arrested, the ends brought together by sutures, with the aid of decalcified bone plates, bobbins or tubes and the wound dressed.

(4) In many cases of strangulated hernia the bowel

## Figures and Tables

**Figure f1:**
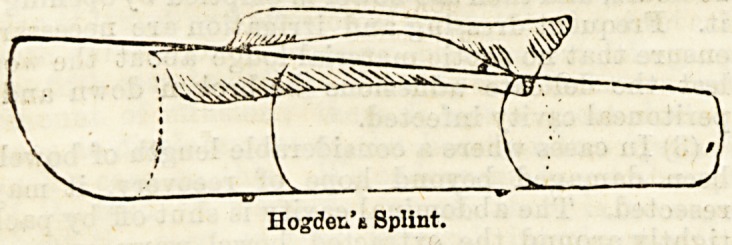


**Figure f2:**
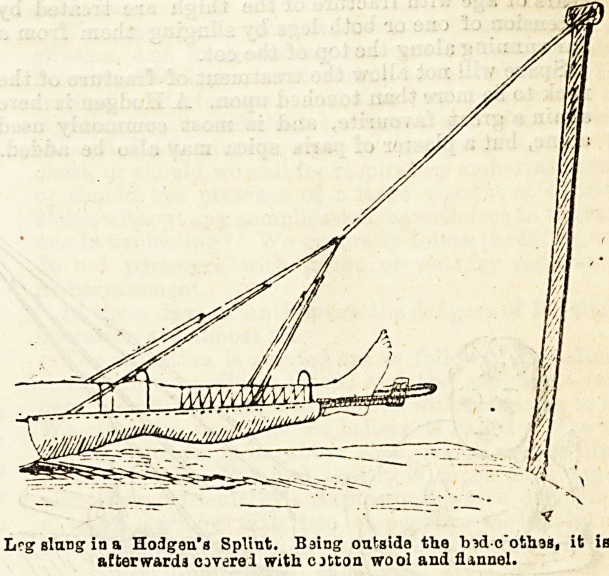


**Figure f3:**